# Meeting the Challenges of COVID-19 in Long-Term Care: Lessons Learned From an International Perspective

**DOI:** 10.1093/geroni/igab046.1573

**Published:** 2021-12-17

**Authors:** Karin Wolf-Ostermann, Marie Boltz

## Abstract

The outbreak of COVID-19 is a major challenge for health care systems all over the world. Older residents of long-term-care facilities (LTCF) such as nursing homes (NH) are among those at highest risk for COVID-19 and comprise a population with dramatically higher rates of morbidity and mortality than the general community. NH staff are also affected by the pandemic as they are challenged by increased workloads, emotional burden caused by the loss of resident life, and the fear of becoming infecting themselves or infecting family members. Finally, the pandemic places emotional and practical demands upon informal carers who are involved in the life of the NH resident. Therefore, research should investigate different perspectives on LTCF during the pandemic and discuss major challenges and possible support structures and strategies. Such an understanding is necessary to optimize care, support post-pandemic recovery, and prepare for future public health challenges. This international symposium will therefore provide four presentations to address these issues. The first presentation will report on global mortality data associated with COVID-19 in LTCF. The second presentation reports on the situation in German NHs addressing the complex situation of morbidity, care dependency, and social isolation. The third presentation will describe the effects of the pandemic upon NH staff in Poland. The final presentation examines the impact and guidelines of allowing visitors in NHs in the Netherlands for residents, family caregivers and staff. Our discussant, Marie Boltz, will synthesize the research findings and lead a discussion of future directions for policy and practice.

